# Assessment of the efficacy of α-lipoic acid in treatment of diabetes mellitus patients with erectile dysfunction: A protocol for systematic review and meta-analysis

**DOI:** 10.1097/MD.0000000000022161

**Published:** 2020-09-04

**Authors:** Jiawei Cai, Junmin Chen, Qianqian Zeng, Jie Liu, Yanli Zhang, Haiping Cheng, Shasha Yao, Qiu Chen

**Affiliations:** Hospital of Chengdu University of Traditional Chinese Medicine, Chengdu, Sichuan, China.

**Keywords:** α-lipoic acid, diabetes mellitus erectile dysfunction, protocol, systematic review

## Abstract

**Background:**

Diabetes mellitus with erectile dysfunction (DMED) is one of the most common causes of disability in diabetic population, and its pathogenesis is related to a variety of factors. Because its pathogenesis is complex and the existing treatment methods have limitations, DMED is difficult to treat in clinical. Recently, some studies have shown that α-lipoic acid (ALA) is associated with DMED, but there is no systematic review and meta-analysis on the relationship between ALA and DMED.

**Methods:**

We will search each database from the built-in until July 2020. The English literature mainly searches Cochrane Library, PubMed, EMBASE, and Web of Science, while the Chinese literature comes from CNKI, CBM, VIP, and Wangfang database. Simultaneously we will retrieve clinical registration tests and grey literatures. This study only screen the clinical randomized controlled trials (RCTs) about ALA for DMED to assess its efficacy. The 2 researchers worked independently on literature selection, data extraction, and quality assessment. The dichotomous data is represented by relative risk (RR), and the continuous is expressed by mean difference (MD) or standard mean difference (SMD), eventually the data is synthesized using a fixed effect model (FEM) or a random effect model (REM) depending on whether or not heterogeneity exists. Erectile dysfunction (ED) will be diagnosed by the International Index of Erectile Function 5 (IIEF-5) score. Finally, meta-analysis was conducted by RevMan software version 5.3.

**Results:**

This study will synthesize and provide high quality to evaluate the effectiveness of ALA supplementation for the treatment of DMED.

**Conclusion:**

This systematic review aims to provide new options for ALA supplementation treatment of DMED in terms of its efficacy and safety.

**PROSPERO registration number:**

INPLASY202070130.

## 1 Introduction

Erectile dysfunction (ED), defined as the inability to achieve and/or maintain an erection sufficient to permit satisfactory sexual intercourse, is a common complication in men with diabetes and has been shown to have a negative impact on the quality of life of patients of all ages.^[1]^ Compared with non-diabetic patients, ED in diabetic patients tends to occur 10 to 15 years earlier and more severe, associated with poor quality of life and poor response to treatment.^[2,3]^ In addition to diabetes, advanced age is another risk factor for erectile dysfunction.^[4]^ Studies have shown that the overall incidence of ED in patients with diabetes is 3 times higher than that in ordinary people, and is higher in men whose average age is more than 60 years old.^[4,5]^ A meta-analysis of 145 studies evaluating the prevalence of erectile dysfunction in 88,577 men with diabetes showed that the overall prevalence of erectile dysfunction in men with diabetes was more than 50%. This was significantly higher in people with type 2 diabetes compared to those with type 1 diabetes.^[6]^ According to statistics, 40% of diabetics over the age of 60 have complete ED.^[7]^ Studies have confirmed that diabetes has a greater effect on permanent ED than intermittent ED.^[8,9]^ In fact, ED usually indicates potential vascular lesions, represents early symptoms of systemic atherosclerosis, and is an independent predictor of cardiovascular events and mortality from various causes.^[10–12]^

Cross-sectional studies have shown that diabetes mellitus with erectile dysfunction (DMED) is associated with advanced age, diabetes, hypertension, hyperlipidemia, smoking, and other diabetic complications.^[10]^ Its pathogenesis is not clear, which is mainly related to the synergistic action of vascular, neurological, and endocrine abnormalities.^[13]^ It is generally believed that DMED is secondary to diabetic neuropathy. Oxidative stress is considered to play an important role in the pathogenesis of diabetic neuropathy.^[14]^ A lot of studies have pointed out that serious oxidative stress occurs in patients with diabetes mellitus (DM), which leads to pathological changes of cavernous smooth muscle, penile erection-related nerves, and blood vessels.^[15]^ The limitation of erectile function is related to increased vasoconstriction and reduced nitric oxide (NO) availability in corpora cavernosa.^[16]^ However, neurogenic NO is considered the most important factor for relaxation of penile vessels and corpus cavernous body, which is synthesized and released through the endothelial and autonomic nerves of penile artery and corpus cavernous body.^[17]^ Studies have shown that increased oxidative stress reduces NO bioavailability by promoting NO inactivation and by decreasing NO production. The increase of oxidative stress can not only disturb the Gi-dependent coupling between cell membrane receptors and the activation of eNOS, but also destroy the dimeric structure of eNOS, reduces eNOS gene transcription and affect its function, thus reducing the production of NO. In addition,the production of endogenous inhibitors of eNOS can be accelerated by increased oxidative stress, which as preferential substrates displace the physiological precursor l-arginine from the enzyme, curtail the production of NO and hence blunt endothelium-dependent relaxations/vasodilatations.^[18]^ Musicki and Burnet study exhibits eNOS and nNOS uncoupling and NADPH oxidase upregulation, along with overall increased oxidative stress may be responsible for the chronic decrease of NO bioavailability of penile endothelial cells and neurons, contributing to the progression of DMED.^[19]^ In addition, strong oxidative stress can also promote the development of chronic inflammation and evoke muscle damage.^[20]^ Advanced glycation end products (AGEs) can also affect the occurrence and development of DMED. During chronic hyperglycemia, advanced glycation end products (AGEs) are formed. Molecular studies on human penile tissues have also shown that AGEs formation contributes to ED by quenching nitric oxide. Compared with non-diabetic patients, AGE products had higher levels in corpus cavernous body of diabetic patients, suggesting a tissue-specific effect of the AGEs.^[21]^

α-Lipoic acid (ALA) is a kind of biological antioxidant, which exists widely in nature. It has many clinical properties.^[22]^ ALA can not only be used as an enzyme cofactor,^[23]^ but also participate in the metabolism of glucose^[24]^ and lipids,^[25]^ and act as an antioxidant to promote the regeneration of endogenous antioxidants and repair all kinds of cell damage caused by oxidative stress.^[26]^ Extensive evidence suggests that ALA has potential therapeutic value in lowering glucose levels in diabetic conditions and that the intracellular redox status plays a role in the modulation of insulin action and insulin resistance. For example, Konrad et al^[27]^ have shown that ALA stimulates glucose uptake by redistributing glucose transporters to the plasma membrane and tyrosine phosphorylation of insulin receptor substrate-1. Other animal studies have shown that ALA can increase and improve glucose uptake and glucose tolerance of skeletal muscle by activating AMPK in skeletal muscle, and help to resist insulin resistance.^[28,29]^ In addition, ALA can be used as an antioxidant to scavenge free radicals and chelate metal ions, improve the state of ischemia and hypoxia of nerve tissue, protect vascular endothelial function, and improve microcirculation.^[30]^ Studies have shown that ALA has a certain ability to inhibit the glycosylation of free radicals and can improve vascular endothelial function through eNOS recoupling and increasing the bioavailability of NO.^[31,32]^ Obesity is not only the influencing factor of diabetes, but also the influencing factor of erectile dysfunction.^[33]^ Huerta et al^[34]^ found that ALA supplementation alone may help to promote body weight loss in healthy overweight/obese women following energy-restricted diets in their clinical randomized controlled study. ALA has become a commonly used drug for the prevention and treatment of diabetic neuropathy.^[35]^ It has been shown to improve nerve blood flow, reduce oxidative stress, and improve distal nerve conduction in a rat model of diabetic neuropathy.^[36]^ In Ziegler et al's randomised,^[37]^ double-blind, placebo-controlled, multicenter, double-arm, parallel trial, ALA has been shown to be effective for mild to moderate diabetic sensorimotor polyneuropathy (DSPN).

Overall, the information collected in this paper shows that ALA has potential therapeutic value in the treatment of diabetic erectile dysfunction. However, there have been no large sample size clinical trials to confirm the effect of ALA supplementation in treating diabetes with ED. Thus, the aim of the current study is to conduct a meta-analysis to reveal the efficacy of ALA supplementation in treating DMED.

## 2 Methods

### 2.1 Protocol registration

The systematic review protocol has been registered on the INPLASY website with registration number INPLASY202070130 (https://inplasy.com/inplasy-2020-7-0130/). The protocol is reported according to the Preferred Reporting Items for Systematic Reviews and Meta-analysis Protocols (PRISMA-P) checklist.^[38]^ If there are any adjustments throughout the study, we will fix and update the details in the final report.

### 2.2 Eligibility criteria

#### 2.2.1 Type of study

Take ALA as main treatment, including randomized controlled trials of the control group (effective methods other than α-lipoic acid). Language is limited in Chinese and English. Non-randomized controlled trials, quasi-randomized controlled trials, case series, case reports, and crossover studies will be excluded.

#### 2.2.2 Participants

Men with a history of diabetes who match the Diagnostic Criteria for Diabetes: Refer to the American Diabetes Association (ADA) Diabetes Care Guidelines.^[39]^ The diagnosis is ED after diabetes, and the International Index of Erectile Function 5 (IIEF-5) score is <21. The course of ED is ≥3 months. The patient must be at least 18 years of age. Neuropathy caused by other causes and patients with severe heart disease, liver and kidney dysfunction, mental illness, or a relevant drug allergic history will be not included.

#### 2.2.3 Interventions

Both groups were cured with conventional diabetes treatments recommended by the ADA guidelines, including diet, exercise, hypoglycemic, and lipid-lowering therapies.^[39]^ The experiment group used ALA, with no limit of the dose and frequency of the medicine while the control group applied for simple western medicine, or placebo, or no treatment. However, once the control group had accepted the therapy of α-lipoic acid, the trials will be rejected. In addition, the 2 groups did not take any drugs that interfered with the outcome indicators. The trial period requires more than 1 course of treatment.

#### 2.2.4 Outcomes

The primary outcome measurement will be assessed using the International Index of Erectile Function 5 (IIEF-5) score.

(1) Healing: IIEF-5 score ≥22 points after treatment;
(2) Significant effect: IIEF-5 score <22 points after treatment, score improvement ≥60%;
(3) Effective: IIEF-5 score <22 points after treatment, points improved <60%, but ≥30%;
(4) Invalid: IIEF-5 score <22 points after treatment, score improvement <30%.

The secondary outcome measurement will be assessed according to the ALA syndrome scoring criteria.

(1) Healing: The clinical symptoms and signs of ALA disappear, and the syndrome score is reduced by ≥90%;
(2) Markedly effective: The clinical symptoms and signs of ALA are obviously improved, the syndrome score is reduced by ≥60%;
(3) Effective: ALA clinical symptoms and signs have improved, syndrome points reduced by <60%, but ≥30%;
(4) Invalid: Chinese clinical symptoms and signs have not improved, or even worse, syndrome scores reduced by <30%. Integral variation formula (Nimodipine method: [(pre-treatment score − post-treatment score) ÷ pre-treatment score] × 100%.

### 2.3 Literature searching strategy

We will retrieve each database from the built-in until July 2020. The English literature mainly searches Cochrane Library, PubMed, EMBASE, and Web of Science. While the Chinese literature comes from China National Knowledge Infrastructure (CNKI) database, Wanfang Data Knowledge Service Platform, the VIP information resource integration service platform (cqvip), China Biology Medicine Disc (Sino Med) with a language limitation of English and Chinese. In addition, we will also search Google scholar, Baidu Scholar to find out unpublished researches or other related literature. And above all, the Chinese Clinical Trial Registry (ChiCTR) and ClinicalTrials.gov will also be searched. A manual search will be conducted at the library of Chengdu University of Traditional Chinese Medicine. The search terms used a combination of relevant medical subject headings (MeSH) and free words: “alpha lipoic acid,” “α-lipoic acid,” “diabetic, erectile dysfunction,” “diabetes mellitus, erectile dysfunction,” “diabetes-induced erectile dysfunction,” “diabetes-induced sexual dysfunction,” “erectile dysfunction,” “sexual function,” “sexual dysfunction,” “impotence”. Two authors (JC and JC) will search and screen all the citations independently. The process of search is presented in Table 1.

**Table 1:**
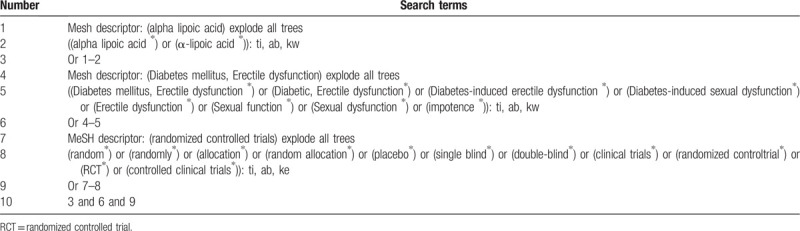
Example of Cochrane search strategy.

### 2.4 Data collection and analysis

#### 2.4.1 Selection of studies

**Figure 1:**
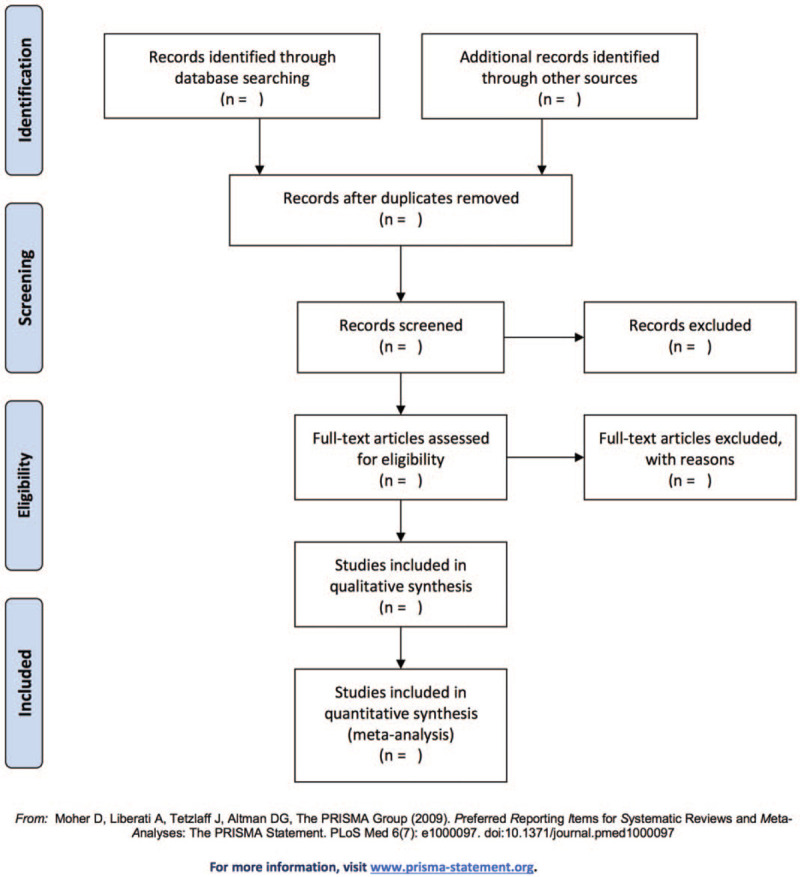
Flow chart of the study selection.

Import all literatures that meet the requirements into Endnote X9 software (Thomson Research Soft, Stanford, Connecticut). First of all, 2 independent reviewers initially screened the literatures that did not meet the pre-established standards of the study by reading the title and abstract. Secondly, download the remaining literatures and read the full text carefully to further decide whether to include or not. Finally, the results were cross-checked repeatedly by reviewers. If there is a disagreement in the above process, we can reach an agreement by discussing between both reviewers or seek a third party's opinion. Flow chart of the study selection (Fig. 1) will be used to show the screening process of the study.

#### 2.4.2 Data extraction and management

According to the characteristics of the study, we prepare an excel form for data collection before data extraction. Outcome indicators for eligible studies were independently extracted and filled in the data extraction form by 2 reviewers. If there is any argument, it can get an agreement by discussing through 2 reviewers or seek a third party's suggestion. For each study, the following data will be extracted: title, the first authors of the article, year of publication, study countries, study design, data collection year, diagnostic criteria used for ED, interventions in experimental group, interventions in control group, time of treatment, course of disease, number of patients in each group, ages of patients, outcomes, and safety data. If there is not enough data in a study, we will contact the corresponding author for more detailed data. If the methodological details are not told in papers, we will contact for more explanation.

#### 2.4.3 Assessment of risk of bias in included studies

Two reviewers will assess the risk of bias of included articles by using the Cochrane Handbook providing the risk of bias (ROB) assessment tool. The following 7 items, such as random sequence generation (selection bias), allocation concealment (selection bias), blinding of participants and personnel (performance bias), blinding of outcome assessment (detection bias), incomplete outcome data (attrition bias), selective reporting (reporting bias), and other bias, are evaluated by 3 grades of “low bias,” “high bias,” and “unclear bias.” Two reviewers will conduct the risk of bias assessment independently and any disagreements will be solved by a discussion of all reviewers.

#### 2.4.4 Measures of treatment effect

Different evaluation methods are selected according to the different efficacy indicators. For the dichotomous data, we will choose the effect scale indicator relative risk (RR) with 95% confidence interval (CI) to represent. While the continuous data is expressed as mean difference (MD) or standard mean difference (SMD) with 95% CI depending on whether the measurement scale is consistent or not.

#### 2.4.5 Dealing with missing data

The reviewers will contact the first author or correspondent author via email or telephone to obtain missing data if the relevant data is incomplete. If the missing data is still not obtained in the above way, we can synthesize the available data in the initial analysis. Furthermore, sensitivity analysis will be used to assess the potential impact of missing data on the overall results of the study.

#### 2.4.6 Assessment of heterogeneity

Heterogeneity will be assessed by chi-squared test and *I*^2^ test. If *I*^2^ < 50%, *P* > .1, we consider that no statistical heterogeneity between each study and choose fixed effect model (FEM) to synthesize the data. If *I*^2^ ≥ 50%, *P* < .1, indicating that there is a statistical heterogeneity, the data are integrated by the random effect model (REM). In addition, due to differences in heterogeneity, we will conduct subgroup or sensitivity analysis to look for the potential causes.

#### 2.4.7 Data analysis

Review Manager software version 5.3 (The Nordic Cochrane Center, The Cochrane Collaboration, 2014, Copenhagen, Denmark) provided by the Cochrane Collaboration will be performed for data synthesis and analysis. The dichotomous data is represented by RR, continuous data is expressed by MD or SMD. If there is no heterogeneity (*I*^2^ < 50%, *P* > .1), the data are synthesized using a fixed effect model. Otherwise (*I*^2^ ≥ 50%, *P* < .1), a random effect model is used to analyze. Then subgroup analysis will be conducted based on the different causes of heterogeneity. If a meta-analysis cannot be performed, it will be replaced by a general descriptive analysis.

#### 2.4.8 Subgroup analysis

If the results of the study are heterogeneous, we will conduct a subgroup analysis for different reasons. Heterogeneity is manifested in the following several aspects, such as race, age, different intervention forms, pharmaceutical dosage form, dosage, treatment course.

#### 2.4.9 Sensitivity analysis

Sensitivity analysis is mainly used to evaluate the robustness of the primary outcome measures. The method is that removing the low-level quality study one by one and then merging the data to assess the impact of sample size, study quality, statistical method, and missing data on results of meta-analysis.

#### 2.4.10 Publication bias assessment

If there are >10 studies in the meta-analysis, the symmetry of the funnel plot will be assessed to examine publication bias, with results being interpreted cautiously.

#### 2.4.11 Grading the quality of evidence

In this systematic review, the quality of evidence for the entire study is assessed using the “Grades of Recommendations Assessment, Development, and Evaluation (GRADE)” standard established by the World Health Organization and international organizations.^[40]^ To achieve transparency and simplification, the GRADE system divides the quality of evidence into 4 levels: high, medium, low, and very low. The GRADE profiler 3.2 will be employed for analysis.

## 3 Discussion

Erectile dysfunction (ED) is very common in men with diabetes and severely affects men's sexual function because patients are often ashamed to speak and thus delay their illness. Now it is getting more and more attention. Drug therapy is the main treatment of erectile dysfunction in diabetes mellitus.^[41]^ Phosphodiesterase-5 (PDE-5) inhibitor is an effective and generally safe drug for the treatment of male erectile dysfunction.^[41,42]^ At present, it is a first-line drug for the treatment of DMED. However, a lot of men with type 2 diabetes do not respond to PDE-5 inhibitors. It is reported that PDE-5 inhibitors have a higher failure rate in diabetic patients than in non-diabetic people.^[43]^ PDE5 inhibitors require minimal nitric oxide production, which is impossible for diabetic neuropathy patients with severe neurological impairment.^[44]^ ALA has a good explanation for this situation and can play a complementary role with PDE-5 inhibitors.

There have been a large number of clinical studies on the treatment of DMED with ALA. However, there is no systematic and comprehensive summary of the existing clinical evidence to evaluate the efficacy of ALA in the treatment of DMED. In this study, we will conduct a systematic review and meta-analysis to provide more evidence-based medicine support for clinical application of ALA supplementation in the treatment of diabetic ED. We will determine the effects of different ages and types of diabetes on efficacy. Finally, there may be some potential deficiencies in this systematic review. Firstly, our study just includes both Chinese and English forms of research, which will lead to the loss of other related studies in other languages. Secondly, it is difficult to obtain indicators for evaluating oxidative stress, which may make a certain bias in the evaluation of the efficacy of ALA.

## Author contributions

**Conceptualization:** Jiawei Cai.

**Data curation:** Jiawei Cai, Junmin Chen.

**Formal analysis:** Junmin Chen, Haiping Cheng.

**Funding acquisition:** Qiu Chen.

**Investigation:** Haiping Cheng, Shasha Yao.

**Methodology:** Jiawei Cai, Jie Liu, Yanli Zhang.

**Project administration:** Qiu Chen.

**Resources:** Jiawei Cai, Qianqian Zeng, Jie Liu, Shasha Yao.

**Software:** Junmin Chen, Qianqian Zeng, Yanli Zhang.

**Supervision:** Qiu Chen.

**Writing – original draft:** Jiawei Cai, Junmin Chen.

**Writing – review & editing:** Qiu Chen.
